# Targeting LRRC41 as a potential therapeutic approach for hepatocellular carcinoma

**DOI:** 10.3389/fmolb.2023.1300294

**Published:** 2023-12-21

**Authors:** Jun Li, Chenjie Qin, Yicheng Wu, Sheng Cheng, Yuanqing Wang, Huijie Chen, Fangli Chen, Bingdi Chen, Jutang Li

**Affiliations:** ^1^ The Institute for Biomedical Engineering and Nano Science, Tongji University School of Medicine, Shanghai, China; ^2^ State Key Laboratory of Systems Medicine for Cancer, Department of Oncology, Shanghai General Hospital, Shanghai Jiao Tong University School of Medicine, Shanghai, China; ^3^ Department of Vascular and Endovascular Surgery, Changzheng Hospital Affiliated to the Naval Medical University, Shanghai, China; ^4^ Hongqiao International Institute of Medicine, Tongren Hospital, Shanghai Jiao Tong University School of Medicine, Shanghai, China; ^5^ Wuxi School of Medicine, Jiangnan University, Wuxi, Jiangsu, China; ^6^ Tongren Hospital, Shanghai Jiao Tong University School of Medicine, Shanghai, China; ^7^ Department of Hematology, Tongren Hospital, Shanghai Jiao Tong University School of Medicine, Shanghai, China

**Keywords:** hepatocellular carcinoma, LRRC41, drug therapy, prognosis, biomarker

## Abstract

**Introduction:** Hepatocellular carcinoma (HCC) is the most common primary liver cancer, characterized by high mortality rate. In clinical practice, several makers of liver cancer, such as VEGFR1, FGFR1 and PDGFRα, were identified and their potentials as a therapeutic target were explored. However, the unsatisfied treatment results emphasized the needs of new therapeutic targets.

**Methods:** 112 HCC patients samples were obtained to evaluate the expression of LRRC41, SOX9, CD44, and EPCAM in HCC, combined with prognosis analysis. A DEN-induced HCC rat model was constructed to verify the expression of LRRC41 and SOX9 in HCC and lung metastasis tissues. Immune score evaluation was analysized by bioinformatics methods. Network pharmacology was performed to explored the potential FDA-approved drugs targeting LRRC41.

**Results:** Through analysis of the Timer database and tissue micro-array, we confirmed that LRRC41 was over-expressed in HCC and exhibited a significant positive correlation with recurrence and metastasis. Immunohistochemistry staining of human HCC tissue samples revealed significant upregulation of LRRC41, SOX9, CD44, and EPCAM, with LRRC41 showing a positive correlation with SOX9, CD44, and EPCAM expression. UALCAN database analysis indicated that LRRC41 and SOX9 contribute to poor prognosis whereas CD44 and EPCAM did not demonstrate the same significance. Furthermore, analysis of a DEN-induced HCC rat model confirmed the significantly elevated expression of LRRC41 and SOX9 in HCC and lung metastasis tissues. Drug sensitivity analysis and molecular docking targeting LRRC41 identified several FDA-approved drugs, which may have potential antitumor effects on HCC by targeting LRRC41.

**Conclusion:** Our findings highlight the role of LRRC41 overexpression in promoting HCC progression and its association with a poor prognosis. Drug sensitivity analysis and molecular docking shows several FDA-approved drugs may be potential therapeutic targets for HCC. Targeting LRRC41 may hold promise as a potential therapeutic strategy for HCC.

## 1 Introduction

Primary liver cancer ranks as the sixth most prevalent and the second most fatal type of cancer (van Meer et al., 2013; Wei et al., 2017; Llovet et al., 2018; Sung et al., 2021). Hepatocellular carcinoma (HCC) and intrahepatic cholangiocarcinoma are different types of primary liver cancer, and HCC accounts for the majority of the cases. The management of HCC involves intricate medical decision-making processes, because of which delayed diagnosis and treatment contribute to high mortality (Llovet et al., 2022). A novel diagnosis and prognostic indicator that could improve clinical outcomes remains elusive (Shi et al., 2021; Dai et al., 2022). Hence, there is a crucial need to explore pathophysiology-related genes in HCC, as they may serve as potential biomarkers for early diagnosis and prognostic prediction.

The leucine-rich repeat containing 41 (LRRC41), a protein-coding gene (Schenkova et al., 2012), is widely expressed across various tissues, such as the thyroid and ovary tissues (The Human Protein Atlas), characterized by two sub-structural domains. Schenkova K et al. have indicated an association between the degradation of LRRC41 protein and the Rho-related BTB (RhoBTB) structural domain-containing protein (Schenkova et al., 2012). However, the function of LRRC41 in HCC progression remains unknown.

Several stemness and progenitor hepatic cell markers have been proven valuable in isolating HCC cells with stem-like properties from hepatocytes (Yamashita and Wang, 2013). These HCC cells with stem-like properties possess the ability for self-renewal, differentiation, and genesis (Liu et al., 2020). Notably, various surface markers have been identified for HCC stem cell subpopulations, including EpCAM, CD133, CD44, CD13, CD90, OV-6, and CD47 (Liu et al., 2020). Additionally, SOX9 has been found essential in the second step of hepatocarcinogenesis in mice (Liu et al., 2022). Furthermore, CD73 upregulates the expression of SOX9 and enhances its stability, thereby playing a crucial role in maintaining stemness and promoting HCC progression (Wang et al., 2020). Specific surface markers like CD44 indicate HCC stem cells (Zarebska et al., 2021), while EpCAM overexpression is associated with poor differentiation and elevated AFP levels in HCC cases (Zhou and Zhu, 2018). Consequently, targeting cancer stem cell markers could offer a promising therapeutic approach (Pang and Poon, 2012). In this study, we established a DEN-induced HCC rat model and found that LRRC41 was significantly upregulated in both HCC and lung metastatic tissues. immunohistochemistry (IHC) staining revealed a significant positive correlation between LRRC41 and SOX9, CD44, and EpCAM. Overexpression of LRRC41 was associated with adverse clinical and pathological manifestations, indicating a potential correlation with SOX9, CD44, and EpCAM. Collectively, these findings emphasized the importance of LRRC41 in providing novel insights for the treatment of HCC.

## 2 Materials and methods

### 2.1 Patient data

A total of 112 HCC samples were obtained from Shanghai Tongren Hospital, and informed consent was obtained from all patients. Supplementary Table S1 provides the baseline information for the clinical samples.

### 2.2 H&E staining & immunohistochemical staining

Hematoxylin and eosin (H&E) staining was conducted to detect pathological changes in liver and HCC tissues. HCC tissue samples were fixed with 4% paraformaldehyde and embedded in paraffin, and immunohistochemical staining was performed as previously described (Jiang et al., 2022). The following antibodies were used: LRRC41 antibody (1:100, bs-8362R, Bioss), SOX9 antibody (1:100, ab185966, Abcam), CD44 antibody (1:100, bsm-51065M, Bioss), and EpCAM antibody (1:100, bsm-52417R, Bioss). The slides were stained with diaminobenzidine tetrahydrochloride (DAB), counterstained with hematoxylin, and images were captured using a Leica microscope.

### 2.3 DEN-induced HCC rat model

Seven week old pathogen-free male Sprague–Dawley rats (weighing 160–180 g, Charles River Laboratories, Beijing) were used in our experiments. All animals were administered diethylnitrosamine (N0756, Sigma) intraperitoneally (70 mg/kg, dissolved in saline) once a week for 10 weeks and sacrificed at 22 weeks (Zhang et al., 2012; Qin et al., 2018).

### 2.4 Immune score evaluation

The RNA sequencing expression profiles (level 3) of HCC and corresponding clinical information were downloaded from the TCGA dataset (https://portal.gdc.com). To evaluate the immune scores reliably of LRRC41 in HCC, we used an R software package that integrates EPIC. Then, Spearman’s correlation analysis of microsatellite instability (MSI) and LRRC41 gene expression was performed. As for the forest plot, the *p*-value, risk coefficient (HR), and univariate analysis of the prognostic characteristics from the single-factor Cox analysis of LRRC41 in tumors were performed. The R software GSVA package was used to analyze the data, employing the “ssGSEA” method (Hanzelmann et al., 2013). Spearman’s correlation analysis was performed to assess the correlations between LRRC41, SOX9, and pathway scores. Univariate Cox regression analyses and forest plots were generated using the “forestplot” R package to present the *p*-value, hazard ratio (HR), and 95% confidence interval (CI) for each variable. All analyses were conducted using R version 4.0.3, and statistical significance was set at *p*-value < 0.05.

### 2.5 Drug sensitivity analysis

Drug sensitivity data were downloaded from the CellMiner website (https://discover.nci.nih.gov/cellminer/home.do). Pearson’s correlation analysis was conducted to investigate the correlation between LRRC41 gene expression and cell sensitivity data of FDA-approved drugs.

### 2.6 Molecular docking analysis

We employed the “query” tool of CMap (https://clue.io/) [20] to screen for chemical compounds with anti-LRRC41 activity. A heatmap was generated to display the top 30 compounds against the LRRC41-related differentially expressed gene signature and their mechanisms of action. For protein–compound interactions, homology modeling of the LRRC41 protein was performed using AlphaFold2 software (Mirdita et al., 2022). The rank1 unrelaxed protein structure was estimated on SAVES v6.0 (SAVES v6.0-Structure Validation Server (ucla.edu)) and used for molecular docking via Discovery Studio software (version 4.5). Auto preparation was applied to prepare LRRC41 and the ligand preparations of compounds. Binding sites and compound conformations were identified, and docking was performed using LibDock. The interaction site, highest LibDockScore, binding pocket 3D view, and intermolecular force distance 2D view were determined. Interaction modes between AZD-5363, temsirolimus, and the top 10 FDA-approved drugs with the LRRC41 catalytic site were visualized using the Discovery Studio Visualizer tool. Molecular dynamics simulations were carried out to investigate the stability of the docking poses of the most potent LRRC41 inhibitor.

### 2.7 Statistical analysis

The relationship between LRRC41 expression and clinicopathological parameters (e.g., age, tumor size, lymph node metastasis, and pathological grading) was tested using the X^2^ test. The SPSS software package version 19.0 was used for the X^2^ test (Wang et al., 2019), with statistical significance set at *p* < 0.05.

## 3 Results

### 3.1 LRRC41 variants, intracellular localization, single-cell variations, and expression profiles under physiological conditions

**FIGURE 1:**
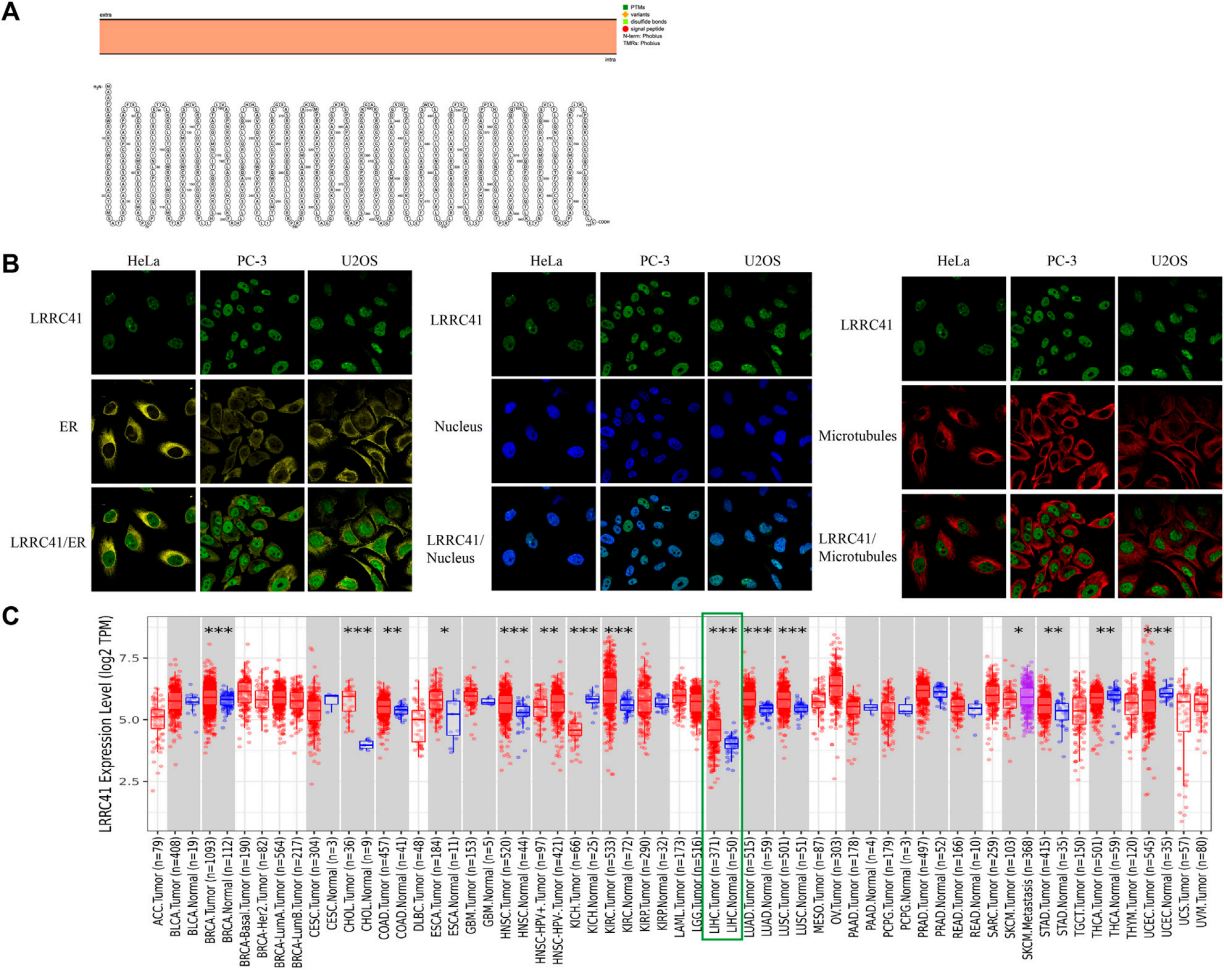
LRRC41 variant, localization, expression profile under physiological conditions, and expression in pan-cancer. **(A)** Protein topology displaying the membrane localization of LRRC41; **(B)** subcellular distribution of LRRC41 from the HPA database; **(C)** overexpression of LRRC41 in hepatocellular carcinoma.

The intracellular membrane localization of LRRC41 was determined and depicted in Figure 1A from Protter—interactive protein feature visualization (ethz.ch). In order to characterize the specific intracellular localization of LRRC41, using the HPA database (Search: LRRC41—The Human Protein Atlas), we were able to obtain immunofluorescence staining images of LRRC41 to examine its distribution within the endoplasmic reticulum (ER) and microtubules of HeLa, PC-3, and U2OS cells. LRRC41 exhibited colocalization with the nuclear marker in HeLa, PC-3, and U2OS cells, suggesting its subcellular localization in the nuclei. Conversely, no colocalization was observed between LRRC41 and the ER or microtubules in these cell lines (Figure 1B). To validate the expression of LRRC41 in HCC, its expression was investigated in pan-cancer samples obtained from the TIMER2.0 (cistrome.org) database. The results indicated that LRRC41 was overexpressed in HCC (Figure 1C).

### 3.2 High expression of LRRC41 in HCC and positive correlation with SOX9, CD44, and EpCAM

**FIGURE 2:**
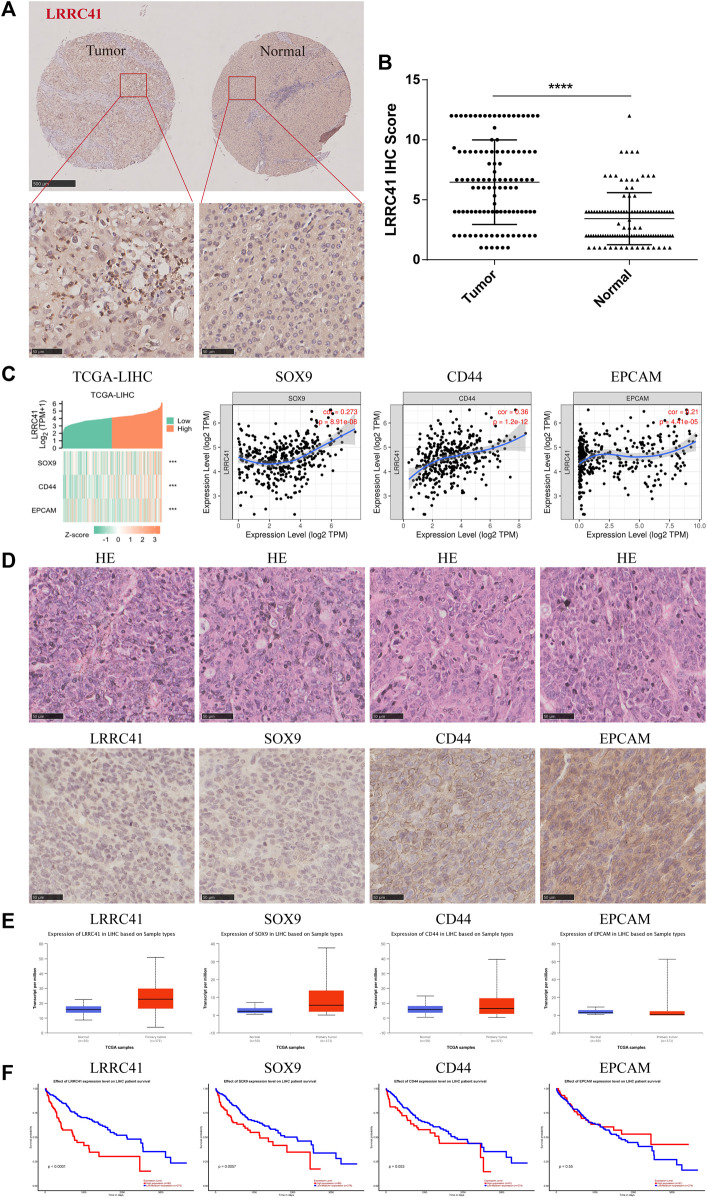
**(A)** Expression of LRRC41 in HCC; **(B)** immunohistochemical scores of LRRC41 in tumor tissues; **(C)** positive correlation between LRRC41 and SOX9, CD44, and EpCAM; **(D)** high expression levels of LRRC41, SOX9, CD44, and EpCAM in HCC tissues; **(E–F)** expression of LRRC41, SOX9, CD44, and EpCAM in HCC samples and their prognostic value on UALCAN(uab.edu).

To explore the relationship between LRRC41 and stemness marker genes, such as SOX9, CD44, and EpCAM in HCC progression, we performed a comprehensive analysis. The TIMER database confirmed the overexpression of LRRC41 in HCC, as shown in Figure 1C, which was further supported by tissue microarray IHC staining using HCC patients’ samples (Figure 2A). Additionally, IHC scores revealed higher levels of LRRC41 in tumor tissues (Figure 2B) (Table 1 and Table 2). Subsequent single-gene correlation analysis results from the TIMER2.0 (cistrome.org) database demonstrated a significant positive correlation between LRRC41 and SOX9, CD44, and EpCAM (Figure 2C). IHC staining results further confirmed the significant upregulation of LRRC41, SOX9, CD44, and EpCAM in HCC samples (Figure 2D). Moreover, the UALCAN (uab.edu) database analysis results demonstrated the significantly high expression levels of LRRC41, SOX9, and CD44 in HCC samples (Figure 2E), with only LRRC41 and SOX9 showing a prognostic value (Figure 2F).

**TABLE 1:** Expression of LRRC41 in HCC and paracancerous tissues.

Expression of LRRC41 protein in HCC tissues and paracancerous tissues			
Tissue	Low expression of LRRC41	High expression of LRRC41	*p*-value
HCC	54	58	0.00003
Paracancerous	92	20	

**TABLE 2:** Patient baseline data sheet: statistical analysis of LRRC41.

HCC tissue			
Characteristic	Low expression of LRRC41	High expression of LRRC41	*p*-value
n	58	54	—
**Age, n (%)**	—	0	—
≥55	25 (%)	20 (%)	0.6444
<55	33 (%)	34 (%)	—
**Sex, n (%)**	—	—	0.6723
Male	50 (%)	44 (%)	—
Women	8 (%)	10 (%)	—
**TRF, n (%)**	—	—	0.0189
Metastasis	10 (%)	21 (%)	—
No metastasis	48 (%)	33 (%)	—
**RR, n (%)**	—	—	0.000001
Recurrence	55 (%)	28 (%)	—
No recurrence	3 (%)	26 (%)	—
**OS, n (%)**	—	—	—
Dead	55 (%)	54 (%)	0.2677
Alive	3 (%)	0 (%)	—
**TTR, n (%)**	—	—	0.51927
YES	38 (%)	27 (%)	—
NO	28 (%)	27 (%)	—

### 3.3 LRRC41 and SOX9 were found significantly overexpressed in HCC and lung metastasis tissues by using the DEN-induced HCC rat model

**FIGURE 3:**
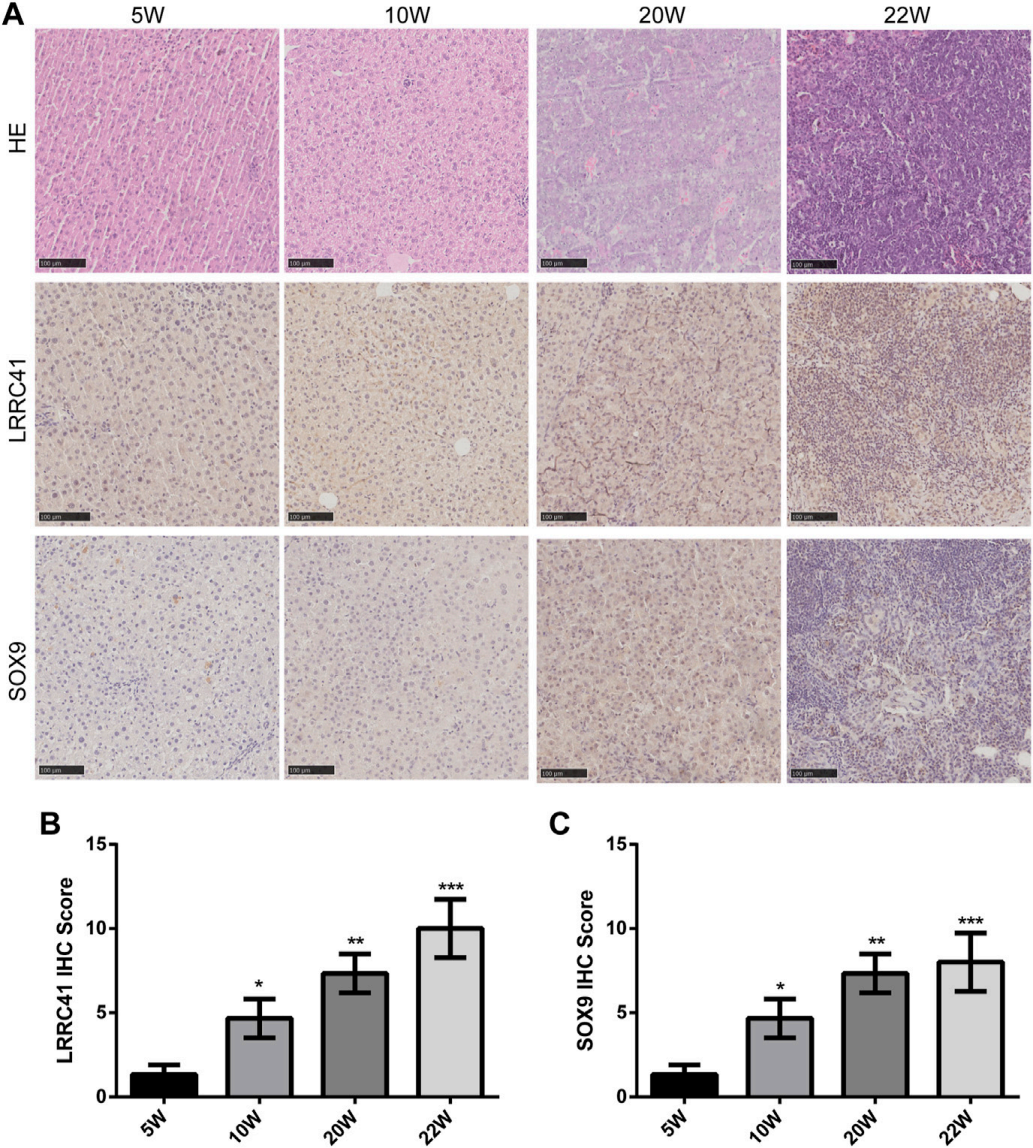
Diethylnitrosamine-induced Sprague–Dawley rat HCC model: **(A)** hematoxylin and eosin staining and IHC staining of LRRC41 and SOX9 in HCC and lung metastasis; **(B,C)** IHC scores of LRRC41 and SOX9 in the DEN-induced SD rat HCC model.

To investigate the role of LRRC41 and SOX9 in the progression of HCC, H&E staining was performed to analyze liver histopathological changes in primary HCC at weeks 5, 10, and 20, as well as lung metastatic foci at week 22. IHC staining was conducted to assess the expression of LRRC41 and SOX9. The results showed that LRRC41 was significantly upregulated in the late stage of the model (Week 20) and hepatocellular lung metastases (Week 22) compared to the early stages of the model (Week 5 and Week 10) (Figure 3A). In addition, the IHC scores of LRRC41 and SOX9 were notably higher at weeks 10, 20, and 22 compared to week 5 of model construction (Figures 3B, C)**.** Collectively, these findings suggest that LRRC41 and SOX9 promote HCC progression.

### 3.4 Establishment and estimation of the prognostic signature

**FIGURE 4:**
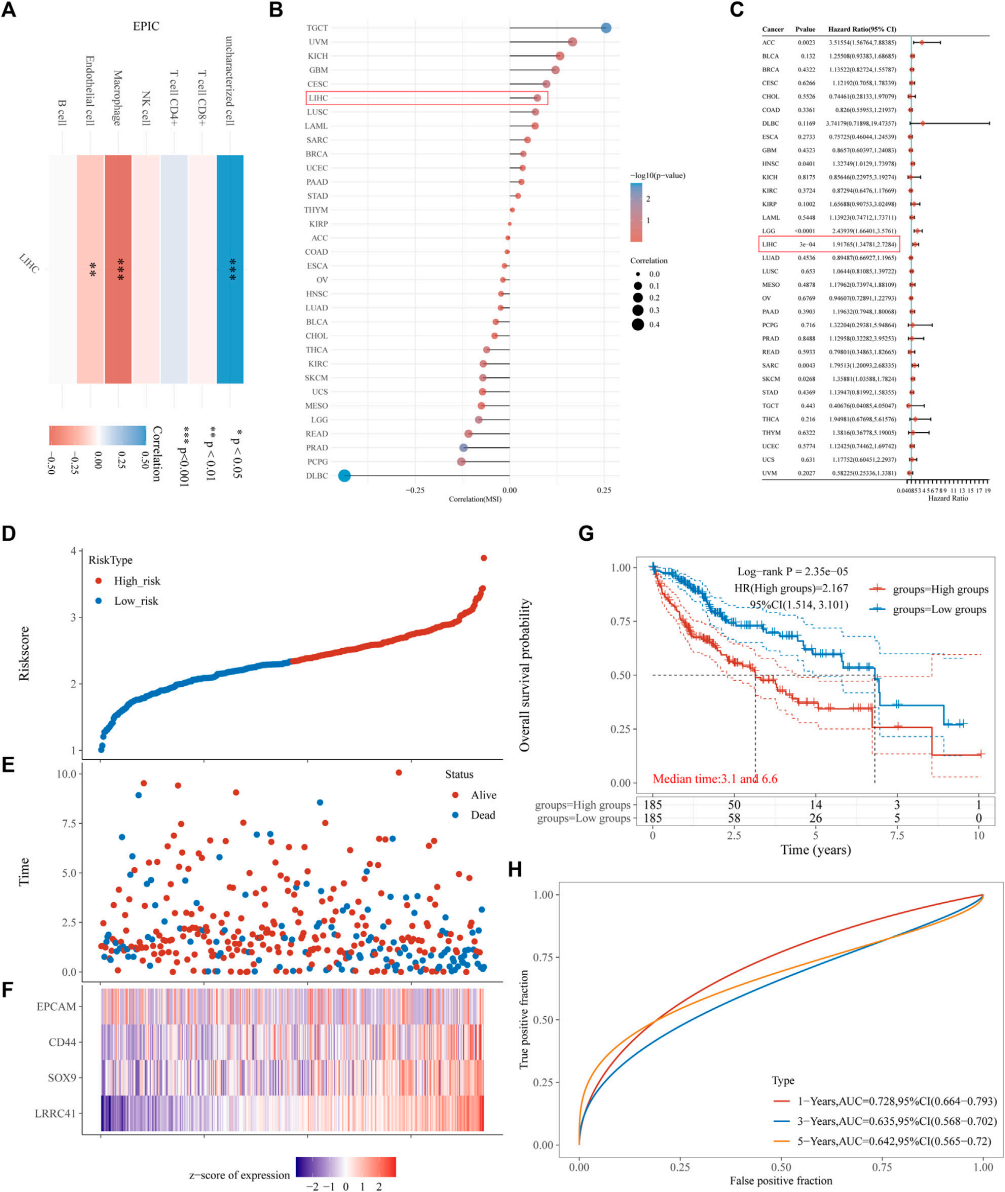
Results of **(A)** heatmap of EPIC immune score; **(B)** microsatellite instability: Spearman’s correlation analysis between MSI and LRRC41 expression; **(C,D)** risk score curves; **(E)** survival status of the patients, with increase in the number of dead patients corresponding to the higher risk score; **(F)** heatmap of the expression profiles of EpCAM, CD44, SOX9, and LRRC41 in the low- and high-risk groups; **(G)** Kaplan–Meier survival curves for the above four-gene signature; **(H)** time-dependent ROC analysis of the four above genes. ROC: receiver operating characteristic.

To evaluate the immune scores of LRRC41 in HCC, an R software package was used to integrate EPIC and the prognostic signature. The RNA sequencing expression profiles (level 3) of HCC and corresponding clinical information were downloaded from the TCGA dataset (https://portal.gdc.com). A heatmap of EPIC immune scores revealed a negative correlation between LRRC41 and macrophages, endothelial cells, and CD8T cells (Figure 4A). Furthermore, Spearman’s analysis confirmed a positive correlation between LRRC41 and MSI (Figure 4B). Univariate Cox regression analyses and forest plots were generated using the “forestplot” R package to present the *p*-value, HR, and 95% CI for each variable. Prognostic characteristics were determined through single-factor Cox regression analysis, demonstrating that LRRC41 had a prognostic value in HCC (Figure 4C). Then, the risk score of every patient was calculated, among which we used the “survminer” R package to obtain the median cut-off point and divided the patients into the high-risk group (n = 185) and low-risk group (n = 185) (Figure 4D **)**. Figure 4E shows the survival status of all patients in the training group, and Figure 4F presents the heatmap of EpCAM, CD44, SOX9, and LRRC41 prognostic genes. The KM survival curves showed that the high-risk group had worse OS than the low-risk group (Figure 4G). Moreover, the EpCAM, CD44, SOX9, and LRRC41 gene prognostic signatures showed larger AUC values in a time-dependent ROC analysis (Figure 4H).

The above evidence indicates that LRRC41 promotes clinicopathological progression in HCC patients, leading to a poorer prognosis.

### 3.5 Drug sensitivity analysis and molecular docking of LRRC41

**FIGURE 5:**
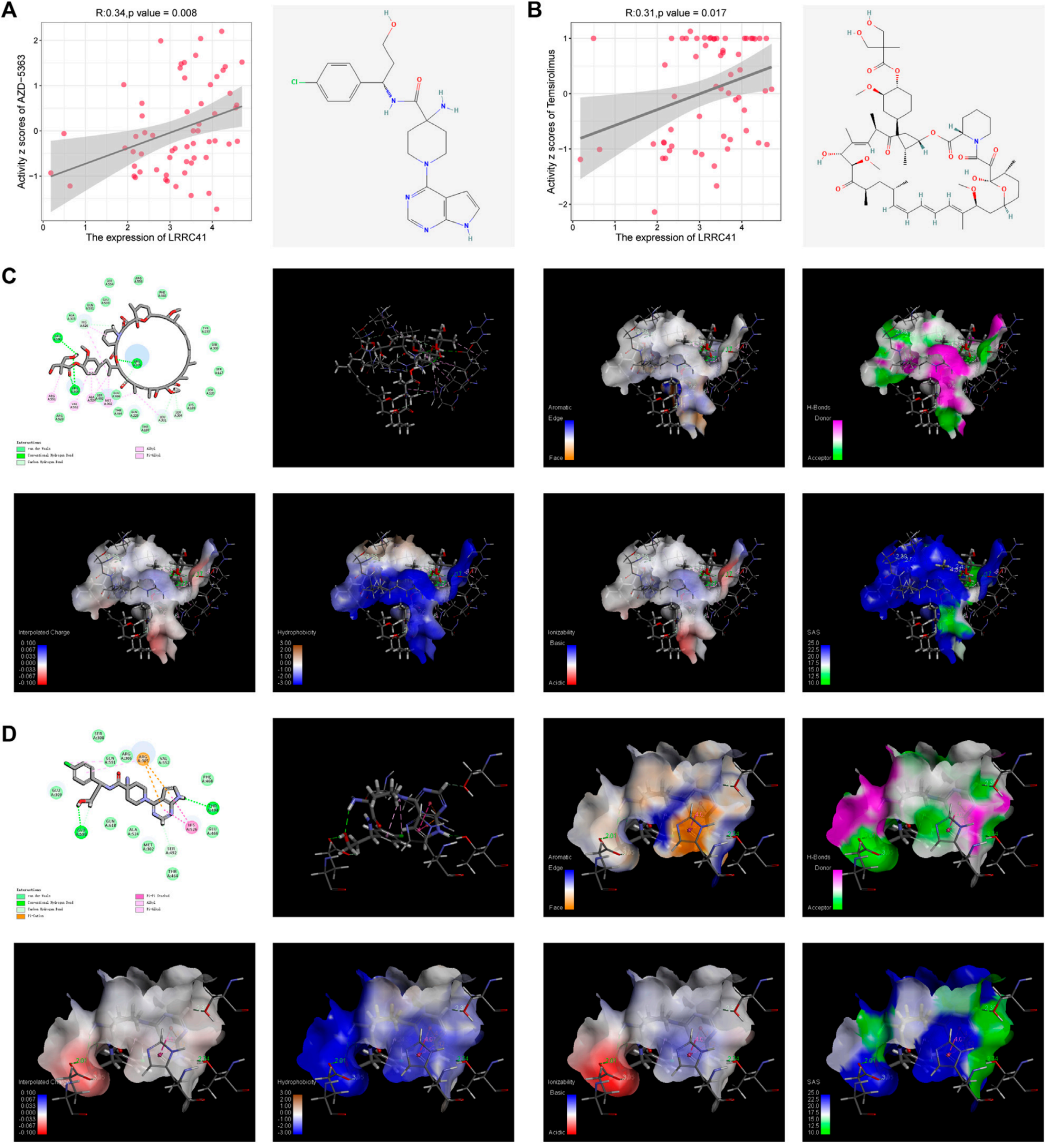
CellMiner analysis of the relationship between LRRC41 and two different compounds: **(A)** AZD-5363 and **(B)** temsirolimus. Molecular docking was performed between LRRC41 and these two compounds, as shown in **(C)** AZD-5363 and **(D)** temsirolimus.

Drug sensitivity data were downloaded from the CellMiner website, and our comprehensive analysis of the drug set data unveiled a significant positive correlation between LRRC41 expression and the activity Z-scores of AZD-5363 (Figure 5A) and temsirolimus (Figure 5B). In order to validate these findings, we performed meticulous molecular docking simulations to assess the binding affinity of LRRC41 with AZD-5363 and temsirolimus. The outcomes of our docking studies demonstrated that LRRC41 exhibited an exceptional geometric and energetic matching pattern with AZD-5363 (Figure 5C) and temsirolimus (Figure 5D), thereby implying the potential efficacy of both drugs in impeding the progression of HCC.

### 3.6 Molecular docking of FDA-approved drugs with LRRC41

**FIGURE 6:**
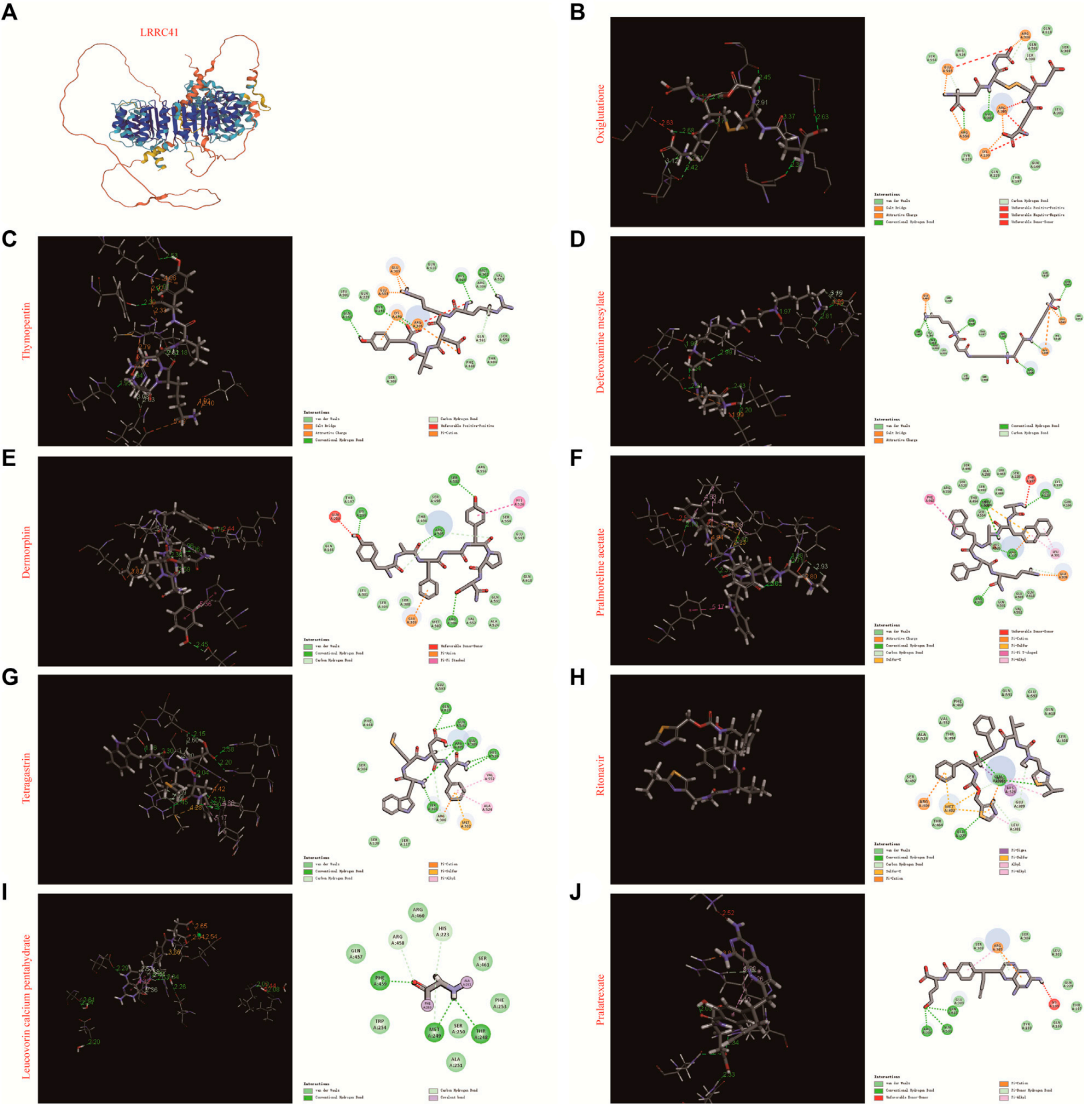
Molecular docking of the top 10 FDA-approved drugs with LRRC4. **(A)** Three-dimensional structure of LRRC41, along with the 3D and 2D structures of docking with **(B)** oxiglutathione, **(C)** thymopentin, **(D)** deferoxamine mesylate, **(E)** dermorphin, **(F)** pralmorelin acetate, **(G)** tetragastrin, **(H)** ritonavir, **(I)** leucovorin calcium pentahydrate, and **(J)** pralatrexate.

**FIGURE 7:**
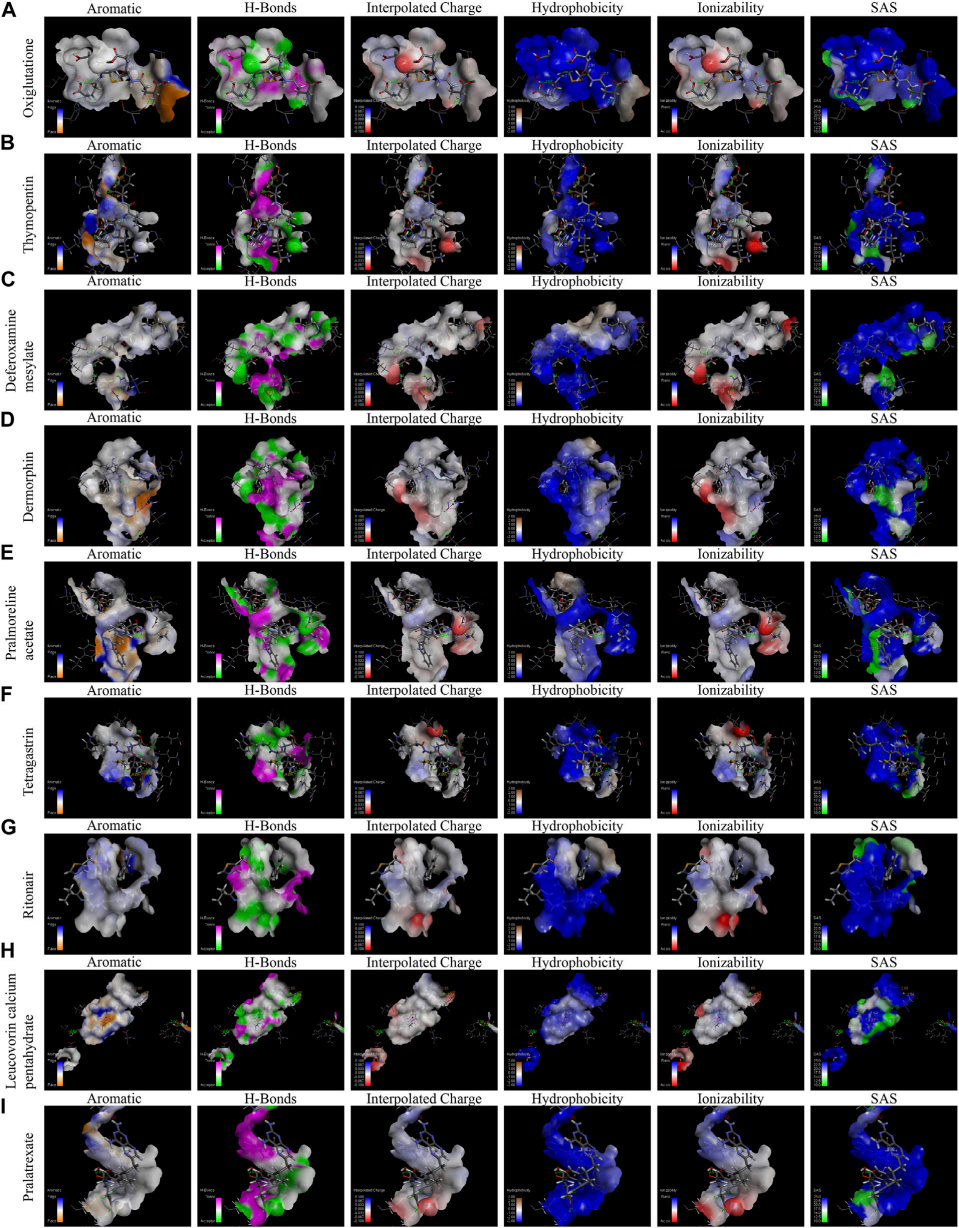
Molecular docking, displaying the binding pocket in a 3D view and the distance of intermolecular forces between LRRC41 and the top 10 FDA-approved drugs. The following features were analyzed for each compound **(A–I)**: aromatic interactions, H-bonds, interpolated charge, hydrophobicity, ionizability, and solvent accessible surface of oxiglutathione, thymopentin, deferoxamine mesylate, dermorphin, pralmorelin acetate, tetragastrin, ritonavir, leucovorin calcium pentahydrate, and pralatrexate.

In order to identify whether those drugs can target LRRC41 or not for HCC treatment, we performed molecular docking studies using the FDA-approved DrugBank. Initially, we assessed all FDA-approved small-molecule drugs based on their topology and selected 300 drugs with energies above 140 kcal/mol for further analysis. Subsequently, 1,800 isomers were identified by analyzing these 300 small-molecule drugs. The LibDock method was employed to analyze the binding affinity between these 1,800 isomers and LRRC41, resulting in the identification of ten drugs with the lowest C-binding energy. Remarkably, LRRC41 exhibited favorable binding with nine out of ten selected small-molecule drugs. Figure 6 shows the 2D and 3D structures of molecular docking; highlighting the aromatic, H-bonds, interpolated charge, hydrophobicity, ionizability, and solvent accessible surface (SAS) properties (Figure 7). These findings suggest that drugs such as oxiglutathione, thymopentin, deferoxamine mesylate, dermorphin, pralmorelin acetate, tetragastrin, ritonavir, leucovorin calcium pentahydrate, and pralatrexate may have potential antitumor effects on HCC by targeting LRRC41, which opens up new possibilities for repurposing existing drugs for the treatment of HCC.

## 4 Discussion

HCC is one of the most common malignancies and a leading cause of cancer-related death worldwide (He et al., 2023). Treatment modalities, such as immune checkpoint therapy (Sprinzl and Galle, 2017; Rotte et al., 2018) and heterogeneous and individualized treatment (Burrell et al., 2013) for liver cancer, are now available, but primary liver cancer, mostly hepatocellular carcinoma, remains a difficult-to-treat problem (Liu et al., 2015). The pathogenesis and precise molecular mechanisms of HCC remain unknown (Facciorusso et al., 2016). In the early stages of HCC, there is no obvious positive sign, delaying the initiation of the treatment (Friedrich and Zustin, 2010). Once HCC cells metastasize, secondary liver cancer (metastatic hepatocellular carcinoma) is likely to occur. Therefore, it is necessary to find out new diagnostic biomarkers and therapeutic targets for HCC. In this study, we observed significant overexpression of LRRC41 in HCC based on databases, tissue samples, tissue microarrays, and an HCC rat model. There was a significantly positive correlation between LRRC41 and stemness genes such as SOX9, CD44, and EpCAM. Ho et al. identified a CD24/CD44-enriched cell subpopulation within EpCAM cells through single-cell transcriptomics, indicating a novel stemness-related cell subclone in HCC (Ho et al., 2019). Deletion of CD44 significantly inhibited metastasis formation of HCC in Nf2-mutant mice (Gerardo-Ramirez et al., 2023). Furthermore, the importance of SOX9 in HCC prognosis has been verified in several studies. Xu et al. explored the prognostic and diagnostic value of SOX9 in cirrhotic hepatocellular carcinoma HCC (CHCC) and non-cirrhotic hepatocellular carcinoma (NCHCC). They found that high SOX9 expression may aid prognostic evaluation in NCHCC (Xu et al., 2021). Ruzinova et al. demonstrated that high SOX9 expression is superior to that of K19 and EpCAM in predicting prognosis in hepatocellular carcinoma (Ruzinova et al., 2023). It was reported that LRRC41 could serve as a predictor of overall survival in patients with oligodendroglioma (Kamura et al., 2001). However, there have been no further reports on LRRC41 in HCC progression (Schenkova et al., 2012). In our study, LRRC41 and SOX9 showed poor prognosis in HCC. We investigated the potential mechanisms by which LRRC41 promotes HCC progression, revealing a negative correlation between LRRC41 and macrophages, endothelial cells, and CD8T cells, and found that LRRC41 was positively correlated with the classical pathways of HCC progression. This suggests that high LRRC41 expression indicates poor prognosis in HCC patients. In conclusion, our study revealed a significant upregulation of LRRC41 in hepatocellular carcinoma, suggesting its potential role in driving the clinicopathological progression of HCC.

To explore LRRC41 as a viable target for drug development, we conducted drug sensitivity analysis and molecular docking experiments. Targeting LRRC41 with AZD-5363, temsirolimus, oxiglutathione, thymopentin, deferoxamine mesylate, dermorphin, pralmorelin acetate, tetragastrin, ritonavir, leucovorin calcium pentahydrate, or pralatrexate demonstrated an antitumor effect on HCC. Our findings may provide a new horizon on the LRRC41-related therapeutic way of treating HCC.

As we know, the PI3K/Akt/mTOR pathway plays a critical role in regulating cell proliferation and survival (Luo et al., 2021). HCC patients with mTOR pathway mutations demonstrated a trend toward shorter time to progression and overall survival (Kelley et al., 2021). AZD-5363, a kt inhibitor that binds to and inhibits all Akt isoforms, which showed favorable binding with LRRC41 in our study, were tested in a phase-I trial in advanced solid tumors, including HCC(Dean et al., 2018), showing acceptable safety and tolerability profiles. Moreover, the combination of AZD-5363 with FH5363 enhanced autophagy-associated death by inhibiting both Akt and β-catenin pathways in HCC. Similarly, temsirolimus has been widely explored in combination therapy as an mTOR inhibitor (Zhou et al., 2012; Kang et al., 2017; Kelley et al., 2021). Knox, et al. conducted a phase-II trial of bevacizumab/temsirolimus doublet in advanced HCC (Knox et al., 2015). Their results showed an encouraging overall response rate of 19% and median overall survival of 14 months in patients enrolled. Those results suggest a possible correlation between LRRC41 and PI3K/Akt/mTOR pathways in HCC. Figuring out this relationship in HCC may lay a solid theoretical foundation for related drug efficacy and widen their application scenario.

The collapsed balance of high oxidative stress and low antioxidant capacities is one of the important causes of the development and progression of HCC. Oxiglutathione, showing favorable binding with LRRC41 in our study, and its related enzymes, plays an important role in the endogenous antioxidant system in the human body (Han et al., 2023). Although there is no clear conclusion of GSH variation in HCC patients (Sanabria et al., 2016; Shimomura et al., 2017), GSH and its related enzymes seem to play important roles in protecting against HCC progression. According to a report by Hsiao et al., HCC patients who had a lower plasma glutathione peroxidase and glutathione reductase activity before tumor resection had a higher HCC recurrence rate (Hsiao et al., 2021). In addition, they observed a higher oxidized glutathione/oxiglutathione ratio at the pre-resection in recurrent patients compared to non-recurrent patients, suggesting the potential role of glutathione in HCC prognosis. Our result predicted the interaction between LRRC41 and oxiglutathione, which provides a novel perspective for predicting HCC prognosis.

In summary, although LRRC41 holds promise as a prognostic biomarker and therapeutic target for HCC, further investigations are warranted to elucidate the underlying molecular mechanisms regulating LRRC41-mediated HCC progression. Additionally, experimental validation is necessary to explore the potential repurposing of existing drugs for HCC treatment.

## 5 Conclusion

LRRC41 exhibits significant overexpression in HCC, thereby contributing to the clinicopathological progression of the disease and resulting in a poor prognosis for patients. Additionally, LRRC41 has been identified as a potential co-contributor in the progression of HCC, along with the stemness gene SOX9. Furthermore, it is worth noting that FDA-approved drugs hold promise as targeted therapies against LRRC41, presenting a potential avenue for effective treatment of HCC.

## Data Availability

The datasets presented in this study can be found in online repositories. The names of the repository/repositories and accession number(s) can be found in the article/Supplementary Material.
